# Soluble LRP1 is an independent biomarker of epicardial fat volume in patients with type 1 diabetes mellitus

**DOI:** 10.1038/s41598-018-19230-3

**Published:** 2018-01-18

**Authors:** David de Gonzalo-Calvo, Cristina Colom, David Vilades, Andrea Rivas-Urbina, Abdel-Hakim Moustafa, Montserrat Pérez-Cuellar, Jose Luis Sánchez-Quesada, Antonio Pérez, Vicenta LLorente-Cortes

**Affiliations:** 1 Lipids and Cardiovascular Pathology Group, Biomedical Research Institute Sant Pau (IIB Sant Pau), Barcelona, Spain; 2 CIBERCV, Institute of Health Carlos III, Madrid, Spain; 3 Department of Endocrinology & Nutrition, Hospital de la Santa Creu i Sant Pau, Barcelona, Spain; 4 Cardiac Imaging Unit, Cardiology Department, Hospital de la Santa Creu i Sant Pau, Barcelona, Spain; 5 Cardiovascular Biochemistry Group, IIB Sant Pau, Barcelona, Spain; 6 CIBERDEM, Institute of Health Carlos III, Madrid, Spain; 7 Biomedical Research Institute of Barcelona-CSIC, Barcelona, Spain

**Keywords:** Diagnostic markers, Endocrinology

## Abstract

Epicardial adipose tissue (EAT) is a metabolically active tissue intimately associated with metabolic syndrome and cardiovascular disease. Quantification of EAT volume is an interesting clinical tool for the evaluation of cardiometabolic disease. Nevertheless, current methodology presents serious disadvantages. The soluble form of the receptor LRP1 (sLRP1) is a non-invasive biomarker of EAT in general population. Here, we analysed the potential of circulating sLRP1 as biomarker of EAT volume in patients with type 1 diabetes mellitus (T1DM). The study included a well-characterized cohort of T1DM patients without clinical cardiovascular disease (N = 73). EAT volume was assessed by a multidetector computed tomography (MDCT). sLRP1 and panel of inflammatory and endocrine mediators were measured using commercially available ELISA. EAT volume showed a direct association with circulating sLRP1 (β = 0.398, *P* = 0.001) in univariate linear regression analysis. This association was higher than that observed for other potential inflammatory and endocrine biomarkers. Using multivariate linear regression analyses, we demonstrated that the association between EAT volume and circulating sLRP1 was independent of potential confounding factors, including age, sex, body mass index, CRP, HbA1c and LDL-C (*P* < 0.050 for all multivariate linear regression models). In conclusion, sLRP1 is an independent biomarker of EAT in T1DM patients.

## Introduction

Cardiovascular disease is the main cause of death in patients with type 1 diabetes mellitus (T1DM), being the relative risk of death by coronary artery disease (CAD) ten times greater than in the non-diabetic population, especially in women^1,2^. The pathophysiology underlying cardiovascular events in patients with T1DM is still unclear and the relative role of conventional cardiovascular risk factors is not well defined. Unlike Type 2 diabetes mellitus (T2DM), T1DM is mainly characterized by insulin deficiency. Nonetheless, insulin resistance and metabolic syndrome are also common pathological derangements contributing to cardiovascular risk in T1DM patients^3,4^. Epicardial adipose tissue (EAT), the fat depot located between the myocardium and pericardium, is an active tissue involved in the development of metabolic and cardiovascular disease^5^. EAT is increased in T1DM and has been associated with insulin resistance/metabolic syndrome^6^ and higher risk of cardiovascular complications^7^. Thus, assessment of EAT could provide relevant insights into cardiometabolic risk prediction in T1DM.

The impact of EAT in cardiometabolic disease has led to a growing clinical interest in the accurate quantification of this fat depot^8^. However, the evaluation of EAT using current imaging modalities is impractical for large-scale population screening and presents several disadvantages including radiation exposure, limited availability and elevated cost^8^. Thus, blood-based biomarkers emerge as non-invasive and early accessible indicators of EAT.

In previous studies, we have demonstrated that the membrane receptor low density lipoprotein receptor-related protein 1 (LRP1) is upregulated in epicardial fat of diabetic patients^9^, and that its soluble form, sLRP1, is a novel biomarker of EAT volume in general population^10^. Insulin deficiency, hyperglycaemia, insulin resistance and systemic inflammation could alter the expression of LRP1^11,12^. Consequently, the relation of sLRP1 with EAT volume could be altered in patients with T1DM with respect to the general population. On the basis of these previous findings, the aim of current investigation was to test whether sLRP1 concentration is associated with EAT volume in a well-characterized and well-controlled cohort of patients with T1DM.

## Results

The main clinical and analytical characteristics of the study population are shown in Table 1. The age at diagnosis was 24.8 ± 8.7 years. All patients received intensive insulin therapy with basal-bolus regimen (14.3% with insulin pump) and the average daily dose of insulin was 0.66 ± 0.22 IU / kg / day. The overall mean HbA1c throughout the follow-up was 7.20 ± 0.79% and in 60% it was ≤ 7.5% during the follow-up. 29% received hypotensive treatment and 42.9% statins. 35.1% were smokers, 40.3% were overweight and 20.8% were obese.

**Table 1:** Characteristics of the study population.

Variable	N = 73
*Clinical characteristics*	
Age (years)	47.1 ± 8.6
Male (%)	44 (60)
Body Mass Index (Kg/m^2^)	27.0 ± 4.6
Waist circumference (cm)	93.9 ± 13.0
Diabetes duration (years)	22.4 ± 2.1
*Biochemical characteristics*	
HbA_1c_ (%)	7.6 ± 1.1
HbA1c (mmol/mol)	59.5 ± 11.8
Total Cholesterol (mmol/L)	4.8 ± 0.7
LDL-Cholesterol (mmol/L)	2.8 ± 0.6
HDL-Cholesterol (mmol/L)	1.5 ± 0.3
Triglycerides ((mmol/L)	1.0 ± 0.9
NEFAs (mmol/L)	0.5 ± 0.3
CRP (μg/mL)	3.4 ± 6.1
*Potential biomarkers*	
IL-6 (pg/mL)	2.3 ± 2.4
IL-10 (pg/mL)	7.8 ± 7.5
TGF-β (pg/mL)	25.3 ± 3.8
Leptin (ng/mL)	1.6 ± 1.5
Adiponectin (ng/mL)	11.4 ± 5.8
sLRP1 (µg/mL)	4.0 ± 1.4
*Epicardial adipose tissue*	
Epicardial Fat Volume (cm^3^)	80.2 ± 49.0
Epicardial Fat Volume indexed by Body Surface Area (cm^3^/m^2^)	41.4 ± 23.1

Data are presented as mean ± SD for continuous variables and as frequencies (percentages) for categorical variables.

Using univariate linear regression analysis we evaluated the association between the EAT volume and a panel of potential circulating biomarkers in T1DM patients, including sLRP1 **(**Table 2**)**. EAT volume showed a direct association with leptin (β = 0.304, *P*-value = 0.011) and sLRP1 (β = 0.398, *P*-value = 0.001). As expected, an inverse association was observed for EAT volume and adiponectin (β = −0.323, *P*-value = 0.006). A borderline association with transforming growth factor β (TGF-β) was also reported (β = −0.209, *P*-value = 0.082). No association was observed between EAT volume and interleukin (IL)–6 (β = 0.076, *P*-value = 0.539) and IL-10 (β = 0.164, *P*-value = 0.175). It should be noted that the standardized beta coefficients were numerically higher for sLRP1.

**Table 2:** Association between EAT volume and potential circulating biomarkers.

Variable	EAT Volume		
	β	*P*-value	
*Univariate analysis*			
IL-6	0.076	0.539	
IL-10	0.164	0.175	
TGF-β	−0.209	0.082	
Leptin	0.304	0.011*	
Adiponectin	−0.323	0.006*	
sLRP1	0.398	0.001*	
*Multivariate analysis*			R^2^
**Model 1**			
sLRP1	0.238	0.015*	0.507
Age	0.322	0.001*	
Sex	−0.318	0.001*	
BMI	0.297	0.004*	
**Model 2**			
sLRP1	0.228	0.024*	0.522
Age	0.298	0.003*	
Sex	−0.319	0.002*	
BMI	0.257	0.016*	
CRP	0.092	0.363	
HbA1c	0.048	0.619	
**Model 3**			
sLRP1	0.251	0.049*	0.723
Age	0.282	0.015*	
Sex	−0.320	0.003*	
BMI	0.262	0.017*	
CRP	0.100	0.342	
HbA1c	0.049	0.613	
LDL-C	−0.042	0.762	

Data are presented as standardized beta coefficient (β) and coefficient of determination (R^2^).

Then, a multivariate linear regression analysis was performed to study in detail the association between the EAT volume and circulating sLRP1 concentration in T1DM patients. Previous publications reported a close association between cardiac adiposity and age, sex or body composition parameters^13,14^. In order to take into account the potential confounding effect of these variables, the association between EAT volume and sLRP1 concentration was adjusted for age, sex and BMI in model 1. As shown in Table 2, the association between the EAT volume and sLRP1 concentration (β = 0.238, *P*-value = 0.015) remained statistically significant even after adjusting for these clinical characteristics. Possible confounders such as CRP, HbA1c or LDL-C, which are associated or have been previously associated with EAT volume and/or sLRP1 concentration^13,15^, were also entered as independent variables into the model 1 (models 2 and 3). The direct association between the EAT volume and circulating sLRP1 concentration remained statistically significant even after adjusting for these confounding factors (β = 0.228, *P*-value = 0.024 for model 2 and β = 0.251, *P*-value = 0.049 for model 3). Due to the association observed between the EAT volume and the circulating concentrations of leptin and adiponectin in the univariate regression analysis, the clinical relevance of smoking and the association between sLRP1 and statin use^15^, an additional model was performed including these four variables. The association between EAT volume and sLRP1 remained statistically significant even after adjusting by these potential confounding factors (β = 0.238, *P-*value = 0.045).

## Discussion

Here, we demonstrated that: i) circulating sLRP1 is a biomarker of EAT volume in a cohort of well-characterized and well-controlled T1DM patients without clinical cardiovascular disease; ii) the direct relation between the EAT volume and circulating sLRP1 concentration is independent of a number of clinical and metabolic confounders; iii) the association reported between EAT volume and sLRP1 concentration is higher than the observed for other potential biomarkers such as leptin, adiponectin or different inflammatory markers.

Since EAT is a modificable factor associated with a higher cardiometabolic risk, therapeutic strategies targeted to reduce EAT (i.e. weight loss or pharmacological interventions) could be implemented in those T1DM patients with high EAT levels^5^. Thus, identifying T1DM subjects with high levels of EAT could be relevant for patient management. Nonetheless, current evaluation of EAT using imaging techniques is not feasible in large-scale screenings. Supporting previous data on general population^15^, circulating sLRP1 is an independent biomarker of EAT volume in T1DM patients that provides additional information to current standard risk factors or established clinical indicators. Importantly, sLRP1 could be cost-effectively quantified in clinical laboratories with current technology, which facilitates its applicability. The evaluation of sLRP1 could constitute an easily performed test to provide additional information to clinicians for monitoring EAT in diabetic care settings.

The pathological role of sLRP1 in cardiometabolic disease is unclear. Our group has identified the cellular form of LRP1 as a key receptor for cholesterol accumulation in vascular cells^16–19^ and cardiomyocytes^20–22^, and showed that this receptor is strongly upregulated in coronary arteries and myocardium in *in vivo* models^23–25^ and patients^22,26–28^ exposed to cardiovascular risk factors. LRP1 has also the potential to interact with different ligands involved in inflammation^29^ and coagulation^30^. We have also shown that circulating levels of sLRP1 are associated with atherosclerosis in patients with familial hypercholesterolemia^15^. Here, we could only speculate about the physiological or pathological implications of the association observed between EAT volume and sLRP1. sLRP1 may affect the functionality of cellular receptor ligands, and thus, may influence different mechanisms linked to cardiovascular disease, such as lipid metabolism, coagulation, inflammation or extracellular protease activity, among others^31–33^. This is an interesting and unexplored field that deserves future investigation.

The main limitation of the current study is the cross-sectional design. As explained above, we cannot draw causal conclusion about the nature of the association between EAT and sLRP1. Since LRP1 is a ubiquitous expressed receptor we cannot conclude whether the sLRP1 detected in plasma is released exclusively from EAT. Despite the robustness of the associations observed here the sample size precluded the analysis of the different subgroups of patients. Finally, generalization of the results is somewhat limited by the defining characteristics of the type 1 diabetes population. Indeed, the detailed analysis of the association between EAT and sLRP1 in patients with cardiovascular disease should be performed.

In conclusion, our results demonstrated that circulating sLRP1 is a novel surrogate biomarker of EAT volume in T1DM patients. A test based on the circulating concentration of this soluble receptor could be an easily accessible tool for cardiometabolic risk stratification in T1DM patients. Further studies should validate these results in multicentric studies with larger populations.

## Methods

Subjects with T1DM who were diagnosed between 1985 and 1994 and regularly followed up at the Endocrinology and Nutrition Department in the Hospital de la Santa Creu i Sant Pau (n = 130 patients) were offered participation. The exclusion criteria was the presence of clinical cardiovascular disease. Seventy-three patients with T1DM and without established clinical cardiovascular disease agreed to participate and were evaluated. Demographic and anthropometric characteristics, cardiovascular risk factors and clinical history were collected. Venous blood samples were taken after overnight fasting for laboratory determinations. Lipid profile was determined according routine protocols of the Sant Pau Hospital, Spain^34^. The study protocol was approved by The Ethical Committee of Clinical Investigation (CEIC) in Sant Pau’s Hospital (http://www.recercasantpau.cat/investigacio-clinica/ceic/). All subjects gave their written informed consent before participating in the study. All methods were performed in accordance with the relevant guidelines and regulation of CEIC-Sant Pau’s Hospital.

EAT was measured in a contrast-enhanced scan acquired with a 256-slice multidetector computed tomography (MDCT) scanner (Brilliance iCT 256-slice, Philips Healthcare). This scan was prospectively triggered at 75% of the RR interval using from 100 to 120 kV (120 kV in patients with a body mass index > 30 kg/m^2^). After that, MDCT studies were analysed on an off-line workstation. The methodology to calculate EAT was done with dedicated software (OsiriX MD, v 6.5, FDA cleared, Pixmeo) as it follows: first, the upper and lower slice limits of pericardium were manually defined using axial views. Then, EAT was marked in each slice by drawing regions of interest with voxel density between −150 to −30 Hounsfield units (corresponding to adipose tissue). After that, a contiguous 3-dimensional volume render (showing EAT volume) was performed and quantified in cubic centimetres (cm^3^) as well as indexed to body surface area (cm^3^/m^2^).

In addition to sLRP1, a panel of endocrine and inflammatory mediators was measured in frozen plasma samples. All mediators were measured using commercial available ELISA: Adiponectin and leptin (R&D), IL-6, IL-10 and TGF-β (Bender MedSystems), and sLRP1 (USCN Life Science).

The statistical software package SPSS 15.0 for Windows (SPSS Inc.) was used for all statistical analyses. Descriptive statistics were used to characterize the study populations and to analyse the studied parameters. Data were presented as the mean ± SD for continuous variables and as frequencies (percentages) for categorical variables. The normality of the data was analysed using the Kolmogorov-Smirnov test. Variables with skewed distribution were log-transformed prior to use as continuous variables. Univariate and multivariate linear regression analyses were performed to examine the associations between EAT volume and circulating sLRP1. The standardized beta coefficient (β) was used to evaluate the strength of the effect of the independent variables. Data are expressed as the standardized beta coefficient (β). Differences were considered statistically significant when *P*-value < 0.050.
