# Publisher Correction: Soluble LRP1 is an independent biomarker of epicardial fat volume in patients with type 1 diabetes mellitus

**DOI:** 10.1038/s41598-019-53407-8

**Published:** 2019-11-08

**Authors:** David de Gonzalo-Calvo, Cristina Colom, David Vilades, Andrea Rivas-Urbina, Abdel-Hakim Moustafa, Montserrat Pérez-Cuellar, Jose Luis Sánchez-Quesada, Antonio Pérez, Vicenta LLorente-Cortes

**Affiliations:** 1 Lipids and Cardiovascular Pathology Group, Biomedical Research Institute Sant Pau (IIB Sant Pau), Barcelona, Spain; 2 CIBERCV, Institute of Health Carlos III, Madrid, Spain; 3 Department of Endocrinology & Nutrition, Hospital de la Santa Creu i Sant Pau, Barcelona, Spain; 4 Cardiac Imaging Unit, Cardiology Department, Hospital de la Santa Creu i Sant Pau, Barcelona, Spain; 5 Cardiovascular Biochemistry Group, IIB Sant Pau, Barcelona, Spain; 6 CIBERDEM, Institute of Health Carlos III, Madrid, Spain; 7 Biomedical Research Institute of Barcelona-CSIC, Barcelona, Spain

Correction to: *Scientific Reports* 10.1038/s41598-018-19230-3, published online 18 January 2018

In the original version of this Article, the author David de Gonzalo-Calvo was incorrectly indexed. This error has now been corrected.

